# 482. SARS-CoV-2 Prevalence in Feces of Very Young Children, A Longitudinal Study

**DOI:** 10.1093/ofid/ofab466.681

**Published:** 2021-12-04

**Authors:** Lydia M Nashed, Jyoti Mani, Sahel Hazrati, Tiana Richards, Naya Nerikar, Shreeya Ravi, Lisa Mattei, Shira Levy, George Maxwell, Suchitra Hourigan

**Affiliations:** 1 Inova Children’s Hospital, Annandale, Virginia; 2 CHOP, Philadelphia, Pennsylvania; 3 Inova Health System, Falls Church, Virginia

## Abstract

**Background:**

Understanding the disease burden of SARS- CoV-2 in young children has been challenging as the majority are asymptomatic or experience mild symptoms and were rarely tested. SARS-CoV-2 is traditionally detected through respiratory secretions but has also been reported in feces where shedding may continue for weeks after respiratory samples show resolution. We examined the prevalence of SARS-CoV-2 in already collected fecal samples from young children through the pandemic as well as associated demographic factors.

**Methods:**

As part of an ongoing longitudinal microbiome study in Northern Virginia, serial stools samples were collected from infants before and throughout the Covid-19 pandemic. Reverse transcription quantitative-PCR detecting SARS-CoV-2 nucleocapsid gene in the N1 and N2 regions was performed. Penalized logistic regression models were developed to evaluate the association between fecal positivity and potential risk factors.

**Results:**

The overall prevalence of SARS-CoV-2 in infant feces was 1.69 % (13 samples) with a prevalence at delivery, 2, 6, 12 and 24 months of 0, 0, 2.56, 1.96, and 0.85 % respectively. Fecal positivity was first detected 31 days before the reported first case of Covid-19 in Northern Virginia; prevalence rates peaked in September at 4.5% (Figure 1). Only one infant who tested positive was symptomatic with COVID-19 21 days before his stool was collected. Of the 13 positive samples, 8 reported Hispanic ethnicity and 7 reported an essential worker (Table 1). Penalized logistic regression model showed association between Hispanic ethnicity and testing positive (OR 5.04 (95% CI 1.7 – 15.0)) that remained after controlling for the presences of an essential worker (OR 4.7 (95% CI 1.6 – 14.0)).

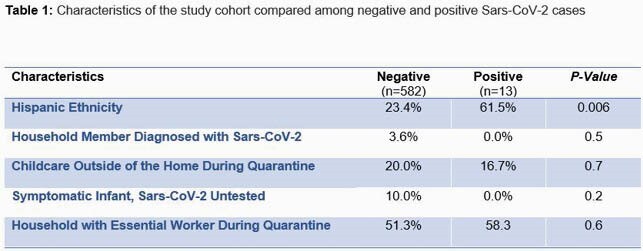

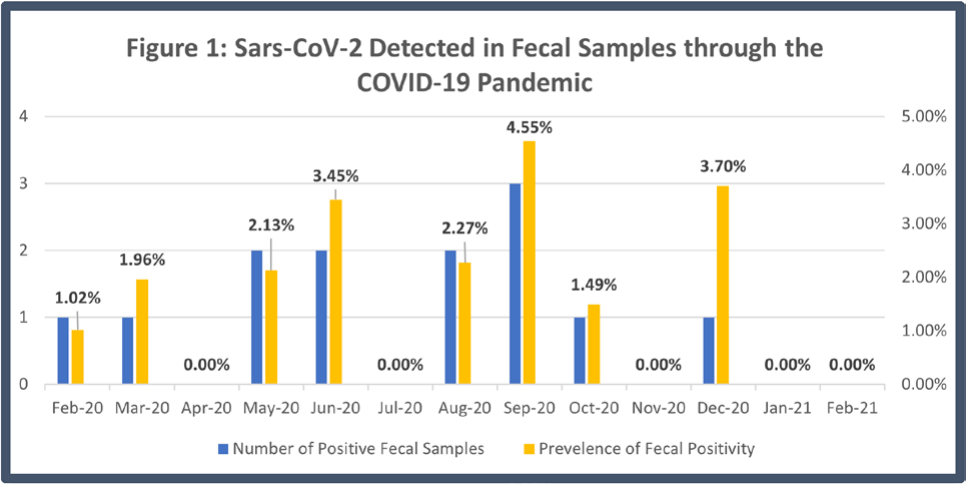

**Conclusion:**

Prevalence of SARS- CoV-2 in infant stool correlated with the prevalence of COVID-19 during the pandemic, with higher rates in those of Hispanic ethnicity corelating with regional trends. Fecal positivity in asymptomatic infants even before quarantine restrictions supports the early but silent transmission of SARS-CoV-2. This study likely underestimates true prevalence rates as stool samples were stored without viral preservative. There are many socioeconomic factors that predispose to disease while ethnicity may be a mediating or confounding factor

**Disclosures:**

**All Authors**: No reported disclosures

